# Acute effect of simultaneous discharge of TASER X26TASER X26 on anesthetized swine model

**DOI:** 10.1186/2197-425X-3-S1-A370

**Published:** 2015-10-01

**Authors:** Y-G Min, E-J Park, S-C Choi

**Affiliations:** Department of Emergency Medicine, Ajou University Hospital, Suwon, Korea, Republic of Korea

## Introduction

The safety of Conducted energy weapons is under debate [1]. The patient's condition and the delivered energy are ones of the affecting factors to the safety [2]. Repeated or long-duration exposure related to the delivered energy can lead to cardiac arrest, apnea, hypotension or academia [3].

## Objectives

The purpose of this study is to investigate the effects of the simultaneous discharge of TASER X26 on anesthetized swine model.

## Methods

This study was a laboratory investigation approved by the animal use committee at our institute. Twelve farm-bred swine weighing between 20 kg and 25 kg were anesthetized with tiletamine/zolazepam and xylazine. The animals were exposed to TASER X26 discharge with one device or two darts (n = 5) in single group and with two devices or four darts (n = 5) in double group. The sham control group (n = 2) were studied using the same protocol as that used for animals in study groups except that they were not exposed to any TASER X26 discharges during the experiments. Hemodynamic parameters were obtained at pre-discharge, at 10, 30 and 60 minutes post-discharge. Blood parameters were obtained at pre-discharge and at 60 minutes post-discharge. Repeated-measures analysis of variance (ANOVA) was performed.

## Results

The significant decrement of systolic blood pressure was shown at 10 minutes after discharge in double group. The decrement of diastolic blood pressure was shown but was not significant at 10 minutes after discharge in double group. The changes of systolic and diastolic blood pressures in double group returned to baseline by 30 minutes after discharge. In single group, the change in blood pressure was not shown. Heart rate and stroke volume index were changed but were not statistically significant in both groups. Serum S100 level, aspartate aminotransferase, and alanine tansaminase were not changed in both groups

## Conclusions

Simultaneous discharge with multiple TASER X26 had an adverse effect on systolic blood pressure in anesthetized swine model.
